# Pictorial essay: Ultrasonography in ‘tennis leg’

**DOI:** 10.4103/0971-3026.73542

**Published:** 2010-11

**Authors:** Jeshil R Shah, Bipin R Shah, Ankit B Shah

**Affiliations:** Saboo Siddique Hospital, Near J. J. Hospital & Acuscan Medical Centre, Juhu, Mumbai, India; 1 Eclate Polyclinic, Ville Parle, Mumbai, India; 2 Life Scan Imaging Center, Malad, Mumbai, India

**Keywords:** Avulsion, gastrocnemius, magnetic resonance imaging (MRI), sonography, tear, Tennis leg

## Abstract

Tennis leg is caused by a rupture of the medial head of the gastrocnemius muscle, usually at its distal musculotendinous junction region. However, tears in this muscle and its tendon are also included under the term ‘tennis leg’. It is seen regularly in practice and is an important cause of a painful calf. The common USG findings include: disruption of the pinnate pattern of the distal medial gastrocnemius, usually near the junction of the triceps surae (which is the echogenic line between the gastrocnemius, the soleus, and the plantaris muscles), fluid tracking along the fascia, adjacent hematoma, and intramuscular tears as well as hematomas. USG is useful for confirming the diagnosis, excluding other causes of a painful calf, for assessing the severity of the disease, and in follow-up.

## Introduction

‘Tennis leg’ is a term that has been in use since 1883, when Powell[1] first described a case. In these patients, the muscle, tendon, and / or the distal musculotendinous junction (MTJ) of the medial head of the gastrocnemius (MHG) is torn. It is usually an avulsion type of injury and is an important cause of a painful calf.[2,3] The spectrum of findings seen in tennis leg are not restricted to tennis players, although it has a high prevalence in this sport.

## Clinical Features

The classical patient is a middle-aged person who complains of a sport-related acute pain in the middle portion of the calf, along with a snapping / popping sensation on extension of the knee and simultaneous forced dorsiflexion of the ankle (i.e., on simultaneous contraction and passive stretching of the gastrocnemius muscle). It can also be caused with sudden activities, for example, when one is running to catch a train or bus, climbing stairs, vehicular accidents, in occasional exercisers (‘weekend warriors’), and so on.[2,3] A few cases have also been reported in association with Namaz praying.[4] Substantial pain and swelling usually develop within 24 – 48 hours.

A painful calf is a common clinical problem. Besides tennis leg, the usual etiologies include deep venous thrombosis, tear of the Achilles tendon, ruptured Baker cyst, arthritis, pyomyositis, abscess, infection, hematoma, bursitis, stress fracture, thrombophlebitis, aneurysm, arteriovenous malformation, compartment syndrome, foreign body, tumor, and so on.[5]

## Anatomical Considerations

Muscles of the posterior compartment of the leg can be divided into superficial and deep crural groups. The superficial crural group (also collectively known as the ‘triceps surae’ / calf muscle complex) includes the gastrocnemius, soleus, and plantaris muscles. The deep crural group includes the tibialis posterior, flexor hallucis longus, and flexor digitorum longus muscles.[6]

The medial head of the gastrocnemius originates from the posterior aspect of the medial femoral condyle, as also that of the lateral head from the lateral femoral condyle. Distally, the MHG merges with the lateral head. Further distally, this merges with the soleus muscle and its tendon to form the Achilles tendon. The plantaris tendon is a small vestigial structure that can be visualized between the MHG and soleus muscles. The gastrocnemius muscle heads have a higher proportion of type II (fast twitch) fibers than does the soleus; these fibers are required for rapid actions such as jumping, running, and so forth. Hence, the heads and distal aponeurosis of the gastrocnemius are common sites of injury and tears.[6–8]

Alhough a few early reports termed isolated ruptures of the plantaris muscle / tendon as ‘tennis leg’, surgical and autopsy studies eventually clarified that the most common lesion causing tennis leg is an avulsion injury of the gastrocnemius — either of the aponeurosis, the tendon, or the muscle itself. Associated soleus injury is also common. An isolated tear of the plantaris tendon is exceedingly rare.[9–11]

## Imaging

Imaging is necessary to rule out other causes of painful calf, to assess the severity of the tear, to prognosticate or advice regarding the period of rest required, during follow-up, to assess healing, and for guiding interventions such as hematoma drainage and steroid and local anesthetic injections.[7,9,12] Other than showing soft tissue swelling, radiographs are noncontributory. USG and MRI are the preferred modalities. USG examination is easily available, quick, painless, economical, and easy to perform. MRI, although not primarily required for establishing the diagnosis, provides a global and extensive view of injuries and even relatively small signal intensity changes can be picked up, unlike with USG. Injuries of other adjacent muscles that are not commonly detected with USG (e.g., injury to the soleus, tibialis posterior, and flexor hallucis longus) are also easily identified with an MRI[6]

## Technique

Eleven patients were examined. All images were acquired using 7-12 MHz electronic linear-array probes on standard scanners [Logiq E and Voluson 730 Expert, both, GE Medical Systems, Bangalore, India], using ample amounts of gel. The patients were placed in the prone position. Comparison was made with the contralateral leg. A small bolster was placed close to the edge of the table, immediately proximal to the ankle and foot, to allow manipulation of the ankle when appropriate (for example, in cases of rupture of the tendo-Achillis — a frequent cause of painful calf). The patient was asked to indicate the point of maximum tenderness. All structures were scanned in the longitudinal and transverse planes, using dynamic imaging. The gastrocnemius is a bulky muscle and produces a distinct bulge over the posterior aspect of the upper leg. The probe was placed over the medial lower belly of the MHG. On a longitudinal view, the MHG is seen well underneath the subcutaneous tissues, and in normal cases, shows a pennate appearance, with hypoechoic muscle fibers separated by the internal perimysium and epimysium.

**Figure 1 (A–C):**
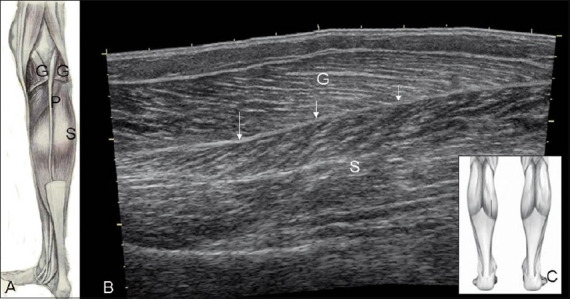
Normal anatomy. Diagram (A) shows the normal position of the vestigial small plantaris tendon (P) between the cut gastrocnemius (G) and soleus (S) muscles. Extended-field-of-view USG image (B), obtained with the probe position indicated in (C) shows the intervening septum or ‘triceps surae’ (arrow).

**Figure 2 (A–F):**
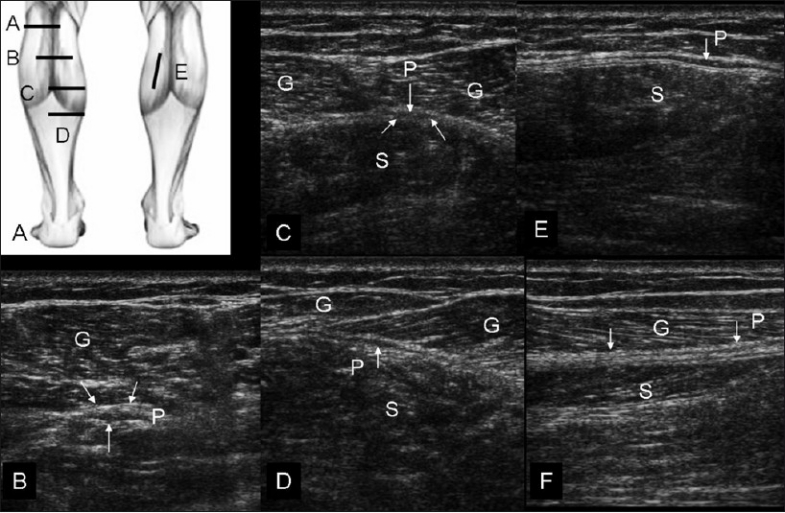
Normal sequential transverse scans of the calf, with the probe positions shown in (A). In position (B), obtained at a relatively high level, the small plantaris (P) muscle is seen below the lateral head of gastrocnemius (G). With progressive distal scans (at levels C, D, and E), the thin tendon of the plantaris (P) (arrows) is seen passing through the triceps surae plane. (F) shows longitudinal view of the plantaris tendon (arrows, P)

It is important to identify the echogenic line (fascial plane) that separates the MHG from the underlying soleus muscle. The thin tendon of the vestigial plantaris muscle also passes through it. This echogenic line is the plane of the triceps surae (formed by the fascial planes of the gastrocnemius, soleus, and plantaris). As the probe is moved distally, it is possible to visualize the MHG muscle transitioning into the muscle tendon junction (MTJ) and the distal aponeurosis. The upper end of the MHG is also examined, with its tendinous insertion into the medial femoral condyle.[7,12] It is difficult to identify the plantaris tendon in all cases. However, on transverse scan, it may be seen medially as a thin ovoid structure between the gastrocnemius and the soleus. It may then be traced proximally up to the lateral femoral condyle and distally up to Achilles tendon, where it lies, adjacent to it [Figures 1 and 2]. Noncompressibility of the adjacent veins with nonvisualization of flow are the criteria used to diagnose venous thrombosis and must always be looked for in these cases.[7,12] In our series, there was no case with associated identifiable deep venous thrombosis (DVT) or demonstrable isolated tear of the plantaris tendon.

## USG Findings of Tennis Leg

**Figure 3 (A–F):**
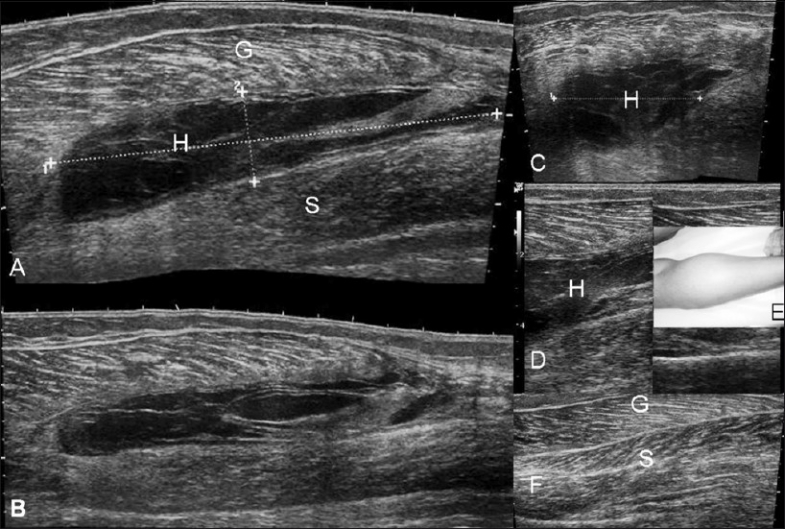
Case 1. Hematoma. Longitudinal, extended-field-ofview (A,B), transverse (C) and longitudinal (D) images demonstrate a large collection / hematoma (H) between the gastrocnemius (G) and the soleus (S). The clinical picture (E) shows the swelling. The normal contralateral USG (F) is shown for comparison See Video 1 at www.ijri.org: Case1. Longitudinal sonography showing internal swirling movements in the hematoma

**Figure 4 (A, B):**
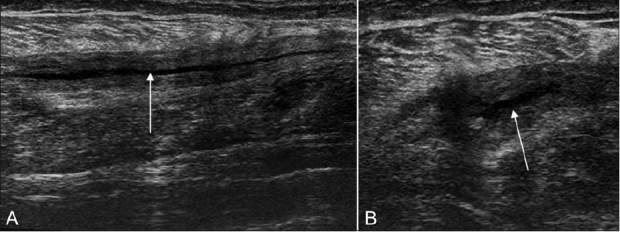
Case 2. Extended-field-of-view longitudinal (A) and transverse (B) images demonstrate a hematoma similar to that in case 1 (although smaller), with thickened walls (arrows); the adjacent muscles show disruption of the normal pennate appearance

**Figure 5 (A–D):**
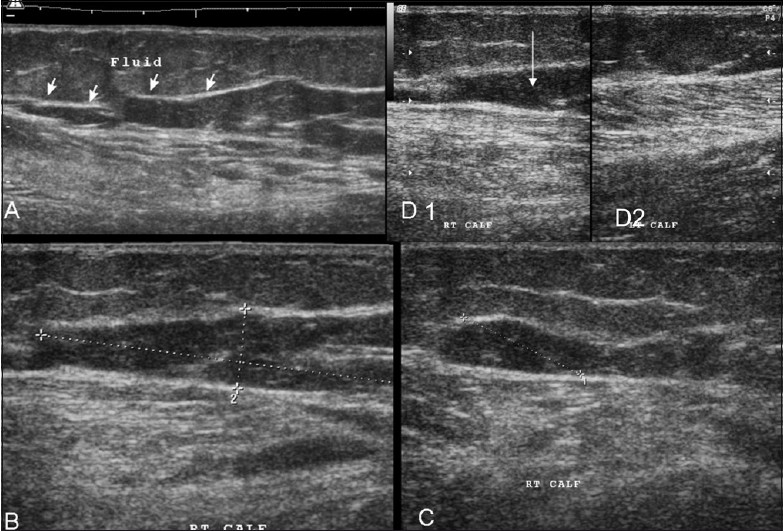
Case 3. Extended-field-of-view longitudinal (A) and standard longitudinal images (B-D1) show a similar hematoma (but with an irregular shape) (arrows); the adjacent muscles show disruption of the normal pennate appearance. Comparison with the normal side (D2) shows that the gastrocnemius has an edematous appearance, with loss of the pennate appearance See Video 2 at www.ijri.org: Video 2: Case 3. Real-time video gives a better perspective of the findings: hematoma & muscle changes.

**Figure 6 (A–C):**
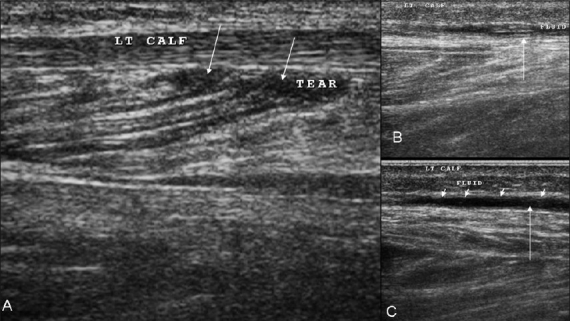
Case 4. Longitudinal images (A–C) demonstrate that there is disruption of the linear intramuscular septae by a small hematoma caused by the tear (arrows in A). Fluid / blood is seen tracking distally along the fascial planes (arrows in B, C)

**Figure 7 (A, B):**
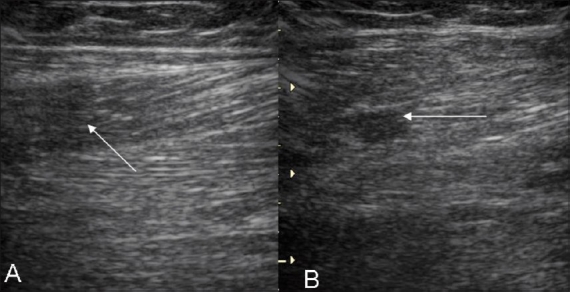
Case 5. Longitudinal (A) and transverse (B) images show a disrupted inhomogeneous pennate appearance and small focal intramuscular hypoechoic areas, suggesting a partial tear (arrows)

**Figure 8 (A–F):**
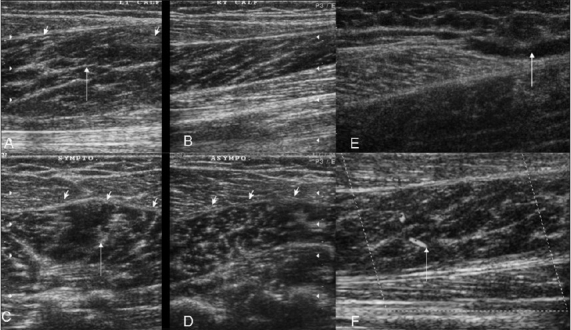
Case 6. Symptomatic (A) and asymptomatic (B) longitudinal and symptomatic (C) and asymptomatic (D) transverse images demonstrate swelling and loss of the normal pennate appearance (arrows) on the left side. Fluid is seen (arrow) near the musculotendinous region on a longitudinal extended field of view image (E). Increased vascularity is seen on the power Doppler (F)

**Figure 9 (A, B):**
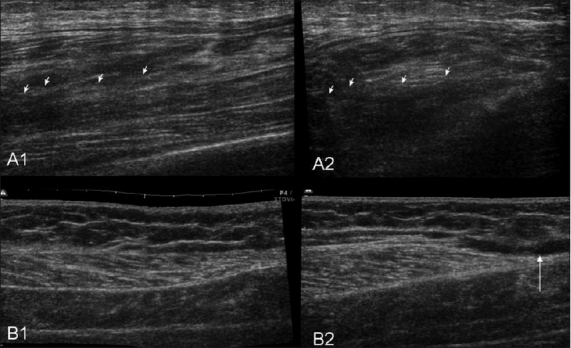
Case 7. Longitudinal (A1) and transverse (A2) images show a partial tear of the gastrocnemius (arrows) with disruption of the pennate pattern and an adjacent hypoechoic hematoma. Case 8: Extended-field-of-view longitudinal (B1) and transverse (B2) images demonstrate fluid (arrow) near the distal aponeurosis

**Figure 10 (A, B):**
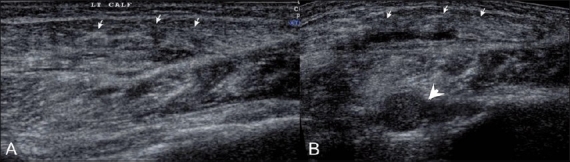
Case 9. Longitudinal (A) and transverse (B) extendedfield-of-view images show marked inhomogeneity of muscles (arrows), with swelling and multiple internal hematoma formations (arrowheads) involving the gastrocnemius and soleus muscles (arrows), in a patient who presented after a vehicular accident

**Figure 11 (A–C):**
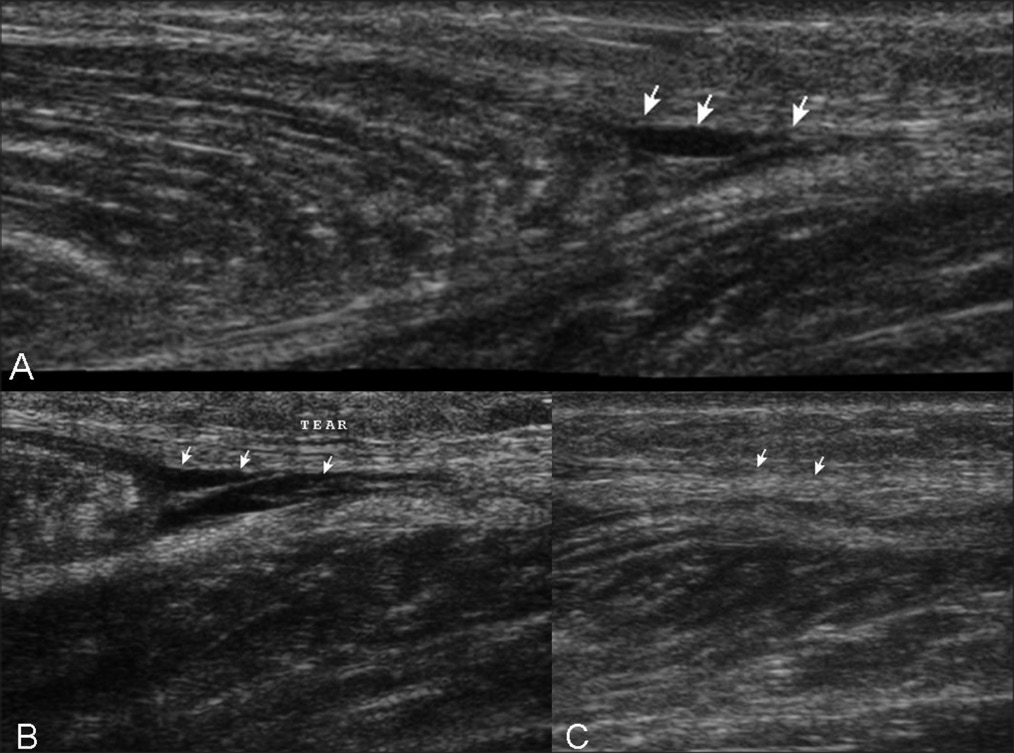
Case 10. Extended-field-of-view longitudinal (A), standard longitudinal (B), and transverse (C) images shows a small hematoma (arrows) near the distal aponeurosis and minimal retraction of the medial head of the gastrocnemius See Video 3 at www.ijri.org: Case 10. A longitudinal sonogram showing small hematoma near the distal aponeurosis See Video 4 at www.ijri.org: Case 10. Transverse sonogram in the same case showing infero-superior sweep with similar findings of hematoma near distal aponeurosis

Diagnosis of partial muscle rupture is based on the presence of localized disruption or discontinuity of muscle fibers, whereas, a complete rupture is defined as a lesion that involves the entire muscle, usually with a gap between the torn ends. On USG, fluid collections / hematomas [Figures 3–5], disruption of the normal pennate appearance of the MHG and / or loss of the fibrillary / echogenic appearance, or an indistinct appearance of the distal MTJ, are common findings[7,12,13][Figures 6–11].

Fluid collections / hematomas in the MHG and the soleus muscles, and extensions along the adjacent fascial planes, the distal aponeurosis, and further distally are usually looked for. A fluid collection as measured by measuring the greatest distance of separation between two muscles determines the prognosis and duration of recovery[7,12] [Figures 3–5]. Tooru[14] divided his series of 29 cases into three types. He suggested that isolated minor tears of the muscle in its belly healed faster than injuries near the MTJ. Prognosis was the worst when a hematoma formed near the MTJ.

**Figure 12 (A, B):**
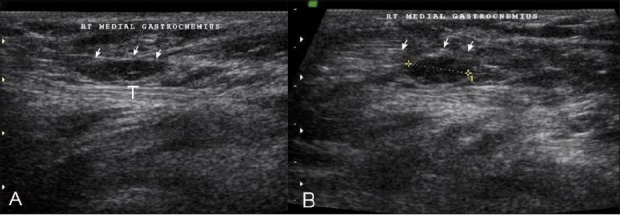
Case 11: Longitudinal USG (A) and transverse (B) images show fluid (arrow) adjacent to the upper end of the gastrocnemius (t), near the medial femoral condyle

One case in our series [Figure 12] presented with pain near the posterior aspect of the medial femoral condyle. Sonography revealed hypoechoic fluid adjacent to the attachment site of the upper tendon of the gastrocnemius. The fluid was not localized, as in bursitis or a small Baker cyst, but was seen spreading into the adjacent soft issues. Subtle inhomogeneities of the tendon were also identified. Partial avulsion of the upper end of the gastrocnemius was diagnosed and the patient was put on conservative treatment. Follow-up after one-and-a-half months revealed complete disappearance of the fluid and resolution of the patient’s symptoms. A Baker cyst, which was less likely to resolve completely was not seen.

Delgado *et al*.,[11] in their series of 141 cases of tennis leg diagnosed with USG, found just two cases of plantaris tendon tear, with visible discontinuity of the plantaris tendon and proximal retraction. Rupture of the MHG was seen in 66% of the patients, collection between the aponeurosis of the MHG and soleus in 21%, soleus rupture in 0.7%, and deep venous thrombosis in 10%.

## Treatment

The RICE protocol (Rest, Ice application, Compression of affected part to prevent swelling and hemorrhage, and Elevation of part) is generally used for conservative treatment. Analgesics and physiotherapy are the other components of conservative management. USG-guided steroid / analgesic injections, as well as hematoma evacuation, are also prescribed in appropriate cases.[6,9,13]

Kvak *et al*.[13] in their series of 30 cases diagnosed with USG, demonstrated that early compressive treatment decreases the amount of hemorrhage and allows early ambulation. Delayed complications of tennis leg include muscle herniation, myositis ossificans, scarring, contractures, functional impairment, and so on.[6,8,9]

**Figure 13 (A, B):**
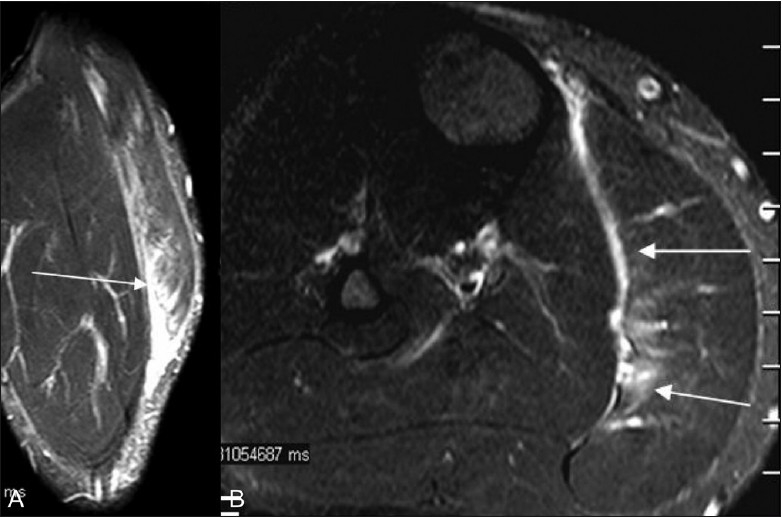
MRI. Coronal T2W (A) and axial fat-saturated T2W (B) images show hyperintensity of the gastrocnemius muscle due to a partial tear (arrows) with fluid along the triceps surae

In conclusion, USG remains a cheap and easily available imaging modality for tennis leg. Higher degrees of tears and hematoma formation are poor prognostic factors, predicting delayed recovery. MRI gives a more global view of the injuries [Figure 13], especially injuries of the muscles other than the gastrocnemius.

### Video available at www.ijri.org

Click here to view as Video 1

Click here to view as Video 2

Click here to view as Video 3

Click here to view as Video 4
